# Anhydrous proton conductor consisting of protamine–monododecyl phosphate composite with self-assembled structure†

**DOI:** 10.1039/d3ra07191j

**Published:** 2023-11-30

**Authors:** Masanori Yamada, Naoaki Yoshihara

**Affiliations:** a Department of Chemistry, Faculty of Science, Okayama University of Science Ridaicho, Kita-ku Okayama 700-0005 Japan myamada@ous.ac.jp

## Abstract

We prepared a protamine–monododecyl phosphate composite by mixing protamine (P) and a monododecyl phosphate (MDP). This P–MDP composite formed an acid–base complex by the electrostatic interaction between cationic protamine and the negatively charged phosphate group. Additionally, according to the X-ray diffraction (XRD) measurements, the composite formed a self-assembled lamellar structure with an interaction between the long alkyl chains of MDP. As a result, the P–MDP composite showed the proton conductivity of 9.5 × 10^−4^ S cm^−1^ at 120–130 °C under anhydrous conditions. Furthermore, the activation energy of the proton conduction of the P–MDP composite was approximately 0.18 eV. These results suggested that the proton conduction of the P–MDP composite was based on an anhydrous proton conductive mechanism. In contrast, the anhydrous proton conduction of the P–methanediphosphonic acid (MP) composite, which did not form the self-assembled lamellar structure, was *ca.* 3 × 10^−5^ S cm^−1^ at 120–130 °C and this value was one order of magnitude lower than that of the P–MDP composite. Therefore, the two-dimensional self-assembled proton conductive pathway of the P–MDP composite plays a role in the anhydrous proton conduction.

## Abstract 2

We prepared an anhydrous proton conductive protamine–monododecyl phosphate composite by mixing protamine (P) and a monododecyl phosphate (MDP).

## Graphical abstract

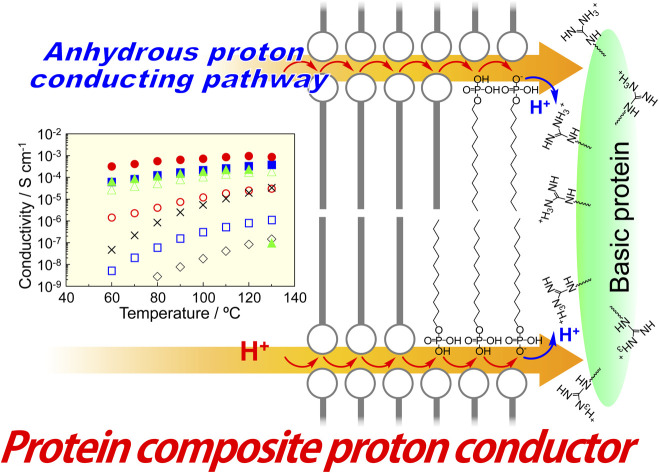

## 1. Introduction

The proton conductor has received attention as an electrolyte membrane of the polymer electrolyte fuel cell (PEFC) without the emission of carbon dioxide. Especially, the PEFC, which is operated at intermediate temperatures (100–200 °C) or under anhydrous conditions, has many advantages, such as a higher energy efficiency, improved CO tolerance of the Pt electrode, fuel management, and co-generation.^1–3^ In contrast, since practical PEFCs have used a humidified perfluorinated sulfonic acid (PFSA) membrane as the proton conducting membrane, at intermediate temperature, the proton conductivity decreased due to the evaporation of the contained water.^1–3^ In addition, the PFSA membranes containing fluorine atoms are expensive because they require many steps to synthesize the PFSA.^4,5^ Furthermore, the PFSA has a high chemical stability and is difficult to dispose of after use. Therefore, electrolyte membranes consisting of a fluorine-free polymer, such as a hydrocarbon polymer or biopolymer, that exhibit the proton conduction at an intermediate temperature under anhydrous or low humidity conditions, have been reported.^6–10^

Recently, we prepared a protamine-acidic molecule composite material and reported its anhydrous proton conduction.^11^ The protamine, one of the proteins, is obtained from fish milt, which is discarded as an industrial waste around the world, and is a strong basic biopolymer containing a large amount of the basic amino acid arginine (Arg).^12,13^ Therefore, a material consisting of protamine can be produced at low cost and its strong basic property can be generated at the proton defect sites in the material. Additionally, since the protamine is a biopolymer, a material consisting of protamine is not only environmentally benign but also biodegradable, making it easy to dispose of after use. Thus, we prepared a composite consisting of the strong basic protein protamine and phosphonic acid and reported its utilization as a proton conductor. As a result, the protamine and methanediphosphonic acid (MP) formed an acid–base complex through the electrostatic interaction between the guanidino group in Arg and the phosphate group in MP and had the proton conductivity of 3 × 10^−4^ S cm^−1^ at 120 °C under anhydrous conditions. This composite material consisting of protamine can be expected to exhibit a higher anhydrous proton conductivity by improving the preparation method of the composite.

On the other hand, proton conductors with the proton conducting pathway in the electrolyte membrane have been reported.^14–20^ These proton conducting pathways are formed by the phase separation of polymers, the surface of nano fiber, the crystalline ion channels, or the self-assembly of surfactant molecules with long alkyl chains.^14–20^ In this case, since the distance between neighboring functional groups is extremely short, the proton transfer easily occurs along the proton conducting pathway. In addition, the proton conductive mechanism in the pathway is a Grotthuss mechanism in which protons transfer from the proton donor site to proton accept site, rather than a vehicular mechanism in which protons move with diffusible vehicle molecules, such as the H_3_O^+^ or H_5_O_2_^+^.^21,22^ As a result, materials with the proton conducting pathway exhibit a high proton conduction under anhydrous or low humidity conditions.^14–20^ Therefore, in this research, we prepared the protamine composite with a two-dimensional proton conducting pathway using self-assembled methods and evaluated its anhydrous proton conduction. The protamine composite with the two-dimensional proton conducting pathway will exhibit a higher anhydrous proton conductivity than the P–MP composite without the self-assembled structure.

In this study, the protamine-monododecyl phosphate (P–MDP) composite was prepared by mixing the protamine (P) and monododecyl phosphate (MDP). These materials formed an acid–base complex through the electrostatic interaction between the guanidino group in Arg and the phosphate group in MDP. According to X-ray diffraction (XRD) measurements, the P–MDP composite formed the self-assembled lamellar structure. Additionally, the phosphate groups in the self-assembled structure behave as a proton conducting pathway. As a result, the P–MDP impregnated glass filter showed the proton conductivity of 9.5 × 10^−4^ S cm^−1^ at 120–130 °C under anhydrous conditions. This proton conductivity was more than one order of magnitude higher than that of the P–methanediphosphonic acid (MP) impregnated glass filter which did not form the self-assembled structure.

## 2. Experimental section

### 2.1. Material

**Scheme 1:**
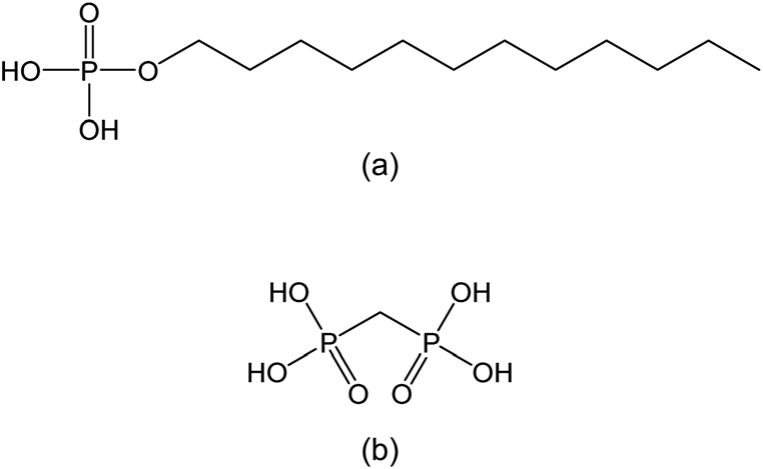
Molecular structures of (a) MDP and (b) MP.

Protamine sulfate (from salmon), monododecyl phosphate (MDP), and methanediphosphonic acid (MP) were purchased from Fujifilm Wako Pure Chemical Industries, Ltd., Osaka, Japan or Tokyo Kasei Kogyo Co., Ltd., Tokyo, Japan. These reagents ware used without the purification. Scheme 1(a) and (b) show the molecular structure of MDP and MP, respectively. The glass filter GC-50 was obtained from Advantec Toyo Kaisha, Ltd., Tokyo, Japan. The thickness of this glass filter was 0.2 mm. The organic solvents were used analytical grade in all the experiments. Ultra-pure water (Merck KGaA, Darmstadt, Germany) was used in all the experiments.

### 2.2. Preparation of P–MDP composite

The purchased protamine sulfate possesses the sulfate ion as a counter ion in its protein. These inorganic ions in the composite can be an obstacle to proton conduction measurements. Therefore, the sulfate ion was exchanged with the hydroxide ion by an ion exchange column using the strongly basic ion exchange resin, Amberlite® IRA-400. The exchange to hydroxide ions was confirmed by a pH measurement. The ion-exchanged aqueous protamine solution was freeze-dried for more than 48 hours and used for subsequent experiments.^11^

The P–MDP composite was prepared as follows: the protamine and MDP were dissolved in ethanol/water (2 : 1, v/v) and ethanol, respectively. In a screw-capped microtube, the MDP solution was added to a protamine solution (50 mg mL^−1^) and the P–MDP mixed solution was heated at 70 °C for 5 minutes. This P–MDP mixed solution was cast on a polytetrafluoroethylene (PTFE) plate and dried overnight at room temperature. These P–MDP composites were used as samples for an infrared (IR), a thermogravimetric-differential thermal analysis (TG-DTA), and an X-ray diffraction (XRD) measurements. In contrast, the samples for the proton conduction measurement were prepared by impregnating a glass filter with the P–MDP composite. This preparation was as follows: the P–MDP mixed solutions were cast on a glass filter (7 × 7 mm^2^) and dried overnight at room temperature.

The molar ratio (*R*) of the MDP in the P–MDP composite was defined by eqn (1):1
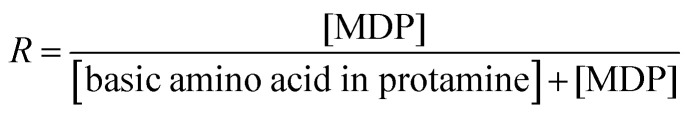
where [basic amino acid in protamine] is the molar concentration of the basic amino acid residues, such as Arg and Lys, in the protamine. According to the amino acid analysis of the protamine, the ratio of Arg was 64.82% and Lys was not detected. [MDP] is the molar concentration of MDP in the P–MDP mixed solution. The *R* value was 0–1. On the other hand, the P–MP composites were also prepared by a similar procedure.

### 2.3. Characterization of P–MDP composite

The molecular structure of the P–MDP composite was determined by the attenuated total reflection (ATR) methods using an FT/IR-4700 infrared spectrophotometer (JASCO Corporation, Tokyo, Japan) with the diamond ATR prism. The IR spectrum was measured at the resolution of 4 cm^−1^. The thermal stability of the P–MDP composite was analyzed by a DTG-60 thermogravimetric-differential thermal analysis (Shimadzu Corp., Kyoto, Japan). The measurement was done at the heating rate of 10 °C min^−1^ under flowing dry-nitrogen. The sample weight was normalized at 1 mg. The X-ray diffraction (XRD) patterns of the composites were measured by a SmartLab (Rigaku Corporation, Tokyo, Japan) or a Philips X'pert (Royal Philips, Amsterdam, Netherlands) at a power level of 45 kV and 200 mA with Cu-K_α_ radiation (*λ* = 1.54 Å) in the 2*θ*/*θ* scanning mode.

### 2.4. Proton conductive measurements of P–MDP composite

The proton conduction of the P–MDP composite was measured as follows: the P–MDP composite impregnated glass filter was sandwiched with two gold electrodes (5 mm diameter).^20^ The electrode samples were placed in a stainless steel vessel, heated at 130 °C for 2 hours under flowing dry-nitrogen to evaporate the water and volatile components, then cooled to room temperature under flowing dry-nitrogen. The proton conductivity of the P–MDP composite was measured by the a.c. impedance method using a 3532-80 chemical impedance analyzer (Hioki Co., Nagano, Japan) from room temperature to 130 °C under flowing dry-nitrogen. The frequency range in the proton conductive measurement was from 4 Hz to 1 MHz.^11,20^ The resistance and proton conductivity of the P–MDP composite was determined from analysis of the Cole–Cole plots.

## 3. Results and discussion

### 3.1. Molecular structure of P–MDP composite

**Fig. 1:**
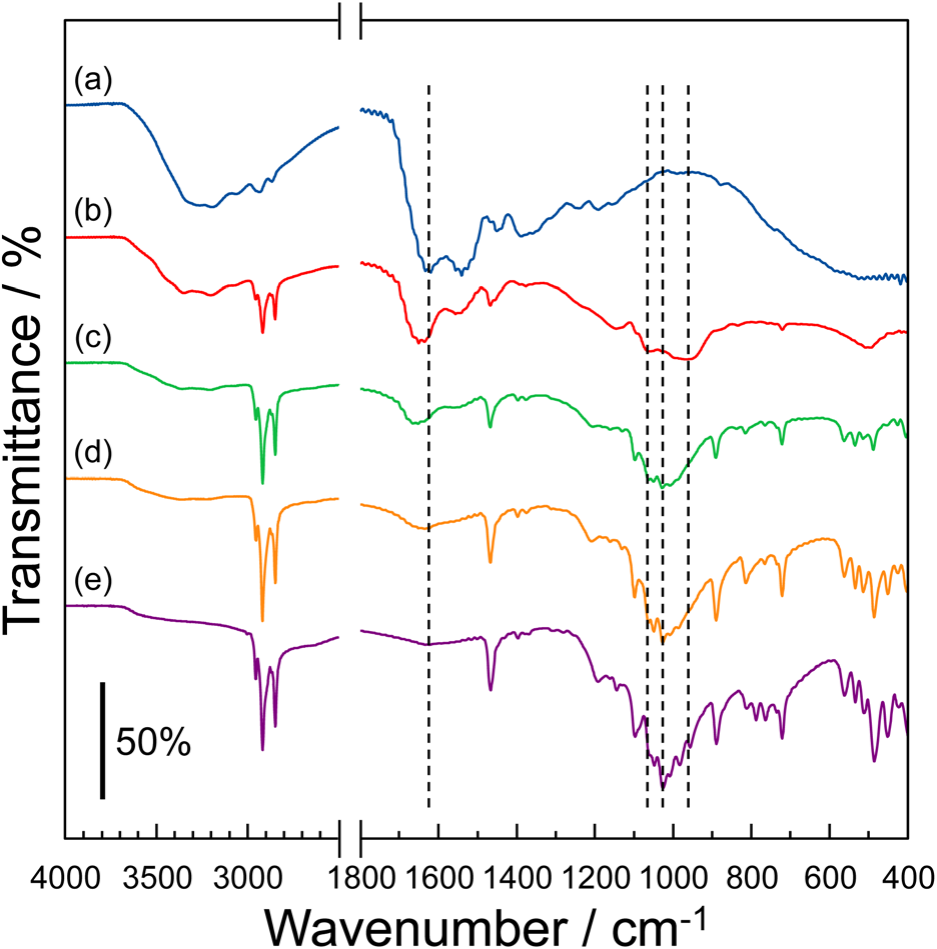
IR spectra of (a) protamine without the composite, (b) *R* = 0.5 composite, (c) *R* = 0.7 composite, (d) *R* = 0.9 composite, and (e) MDP without the composite. Similar results were obtained for triplicate experiments.

The molecular structure of the P–MDP composite was evaluated using an IR spectrometer with the diamond ATR prism. Fig. 1 shows the IR spectra of (a) protamine without the composite, (b) *R* = 0.5 composite, (c) *R* = 0.7 composite, (d) *R* = 0.9 composite, and (e) MDP without the composite. The protamine without the composite had an absorption band at 1620 cm^−1^, related to the stretching vibration of N–H.^11,23–25^ This absorption band decreased by the addition of MDP, and at the same time, a new absorption band appeared at 1637 cm^−1^. Since the absorption band at 1637 cm^−1^ was attributed to the stretching vibration of the –NH_3_^+^ group,^11,23^ the amino groups present in the guanidino group of protamine were protonated by the addition of MDP. In contrast, the MDP without the composite showed an absorption band at 1024 cm^−1^, related to the asymmetric stretching vibration of the P–OH group.^11,23,26,27^ This absorption band relatively decreased with the addition of protamine. Additionally, a new absorption band at 953 cm^−1^ and 1054 cm^−1^, attributed to the deprotonation of the P–OH group,^11,23,26,27^ appeared by the addition of protamine. Consequently, the addition of protamine to the MDP induced the deprotonation of the P–OH group in MDP. These results suggested that the P–OH group in the MDP molecules and the amino site of the guanidino group in the protamine behave as the acidic and basic molecules, respectively, and the protamine and MDP form the acid–base complex through the electrostatic interactions between the P–O^−^ group and the –NH_3_^+^ group. Similar results, such as the complex formation by an electrostatic interaction, have been reported for various anhydrous acid–base proton conductors.^11,20,28^

### 3.2. Thermal property of P–MDP composite

**Fig. 2:**
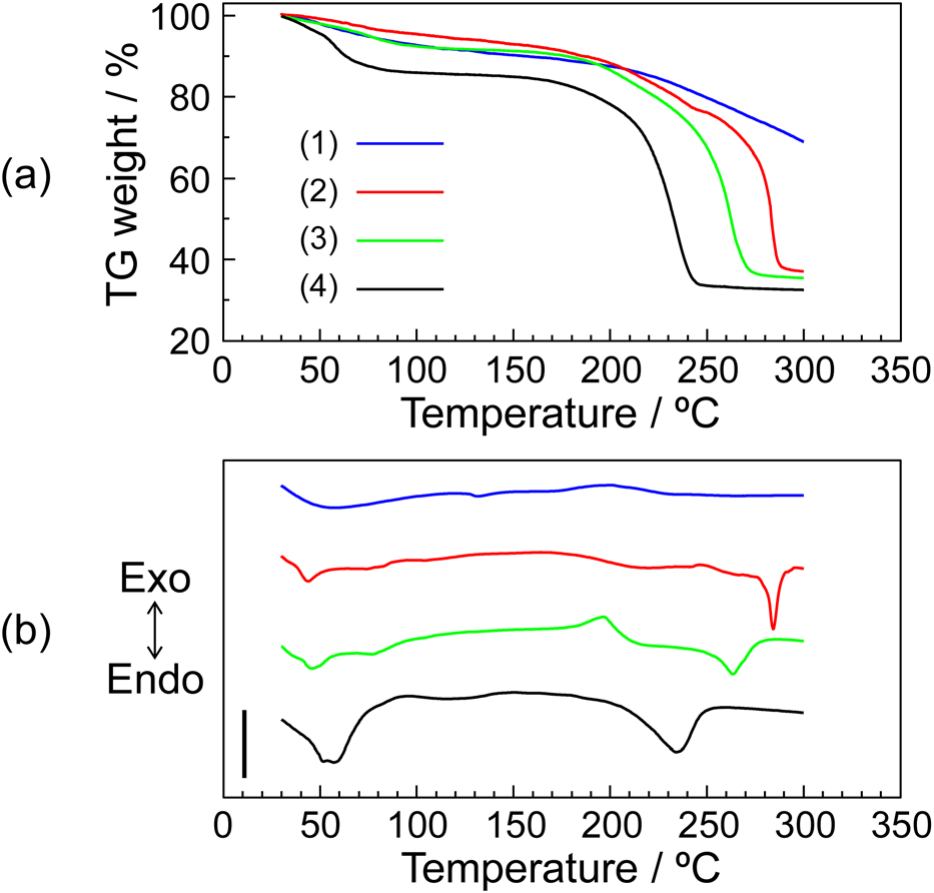
TG (a) and DTA (b) curves of (1) protamine without the composite, (2) *R* = 0.7 composite, (3) *R* = 0.9 composite, and (4) MDP without the composite. The TG-DTA measurements were done at the heating rate of 10 °C min^−1^ under flowing dry-nitrogen. The scale bar in (b) shows 20 μV. Similar results were obtained for triplicate experiments.

The thermal property of the P–MDP composite was evaluated by TG-DTA. These measurements were done at the heating rate 10 °C min^−1^ under flowing dry-nitrogen. The sample weights were normalized at 1 mg. Fig. 2 shows (a) TG curves and (b) DTA curves of (1) protamine without the composite, (2) *R* = 0.7 composite, (3) *R* = 0.9 composite, and (4) MDP without the composite, respectively. All samples showed an endothermic peak at <100 °C. These endothermic peaks were due to the evaporation of water contents and appeared as the TG weight loss at <100 °C. Additionally, in the protamine without the composite, the TG curve showed a further TG weight loss above 200 °C and the DTA curve showed an exothermic peak at approximately 200 °C. These results suggested that the thermal decomposition of protamine without the composite occurred above 200 °C. In contrast, the MDP without the composite exhibited a large endothermic peak and a high TG weight loss at around 200 °C. This is due to the dehydrated reaction of the phosphate group and similar results have been reported.^11,20,29^ This endothermic peak related to the dehydrated reaction was shifted to a higher temperature by the addition of protamine. The reason for these phenomena is as follows: since the guanidino groups in protamine and the phosphate groups in MDP form an acid–base complex through an electrostatic interaction, the addition of protamine increases the distance between the non-deprotonated P–OH groups in MDP. As a result, a higher temperature was required for the dehydration between the distant P–OH groups. Additionally, at high *R* values, such as 0.9 ≤ *R*, an exothermic peak appeared at around 185 °C. This phenomenon can be postulated of as follows: the protons of MDP added in excess behave as acidic catalysts in the composite. As a result, the thermal decomposition of protamine occurred at a lower temperature. In fact, at *R* = 0.95, the exothermic peak appeared at an even lower temperature (see Fig. S1 in the ESI†). These results suggested that although the thermal stability of MDP increased with the addition of protamine, the addition of excess MDP decreased the thermal stability of protamine. Especially, at a high *R* value, the thermal decomposition of protamine occurred at <180 °C.

### 3.3. Proton conductivity of P–MDP composite

The proton conductive samples were prepared by impregnating a glass filter with the P–MDP composite. The proton conductive measurements were done under flowing dry-nitrogen. Additionally, before the measurement, the samples were heated at 130 °C for 2 hours under flowing dry-nitrogen to evaporate the water and volatile components. Therefore, in our proton conductive samples, there are no ions other than the protons. The protamine without the composite did not show any measureable proton conduction. When the MDP was added to the protamine, the proton conduction appeared at 0.6 ≤ *R*. In addition, at high *R* values, the proton conduction abruptly decreased above 130 °C due to the thermal decomposition of the protamine. Therefore, the proton conductive measurements were demonstrated in the range from room temperature to 130 °C.

**Fig. 3:**
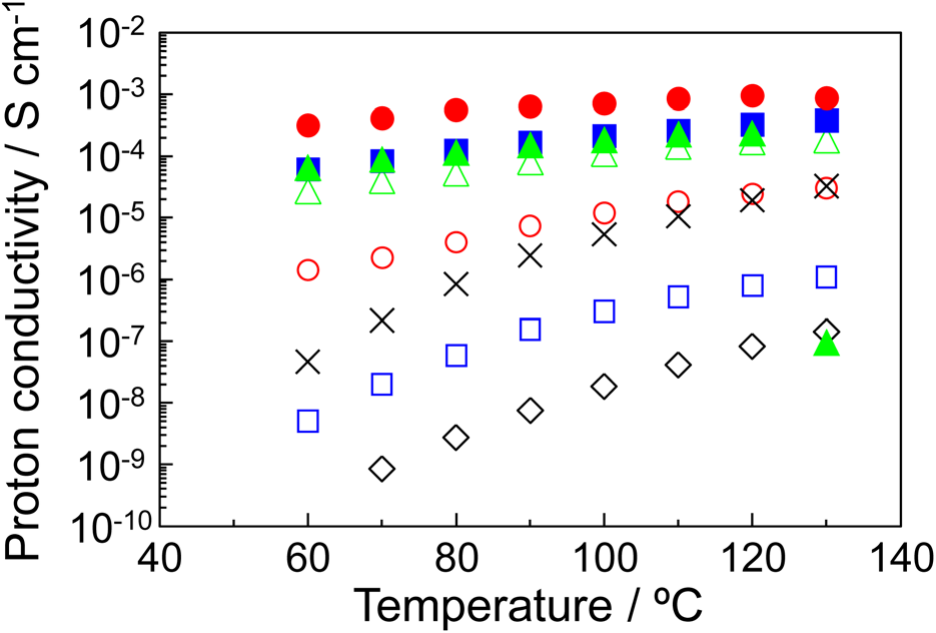
Proton conductivity of the P–MDP composites. The molar ratios are (◇) *R* = 0.6, (□) *R* = 0.7, (◯) *R* = 0.8, (△) *R* = 0.9, (■) *R* = 0.93, (●) *R* = 0.95, and (▲) *R* = 0.98. (✕) shows the P–MP composite with *R* = 0.95. Similar results were obtained for triplicate experiments.

Fig. 3 shows the proton conductivity of (◇) *R* = 0.6, (□) *R* = 0.7, (◯) *R* = 0.8, (△) *R* = 0.9, (■) *R* = 0.93, (●) *R* = 0.95, and (▲) *R* = 0.98 composites under flowing dry-nitrogen (Fig. S2 in the ESI1† shows the proton conductivity of P–MDP composites with its standard deviation.) The proton conductivity increased with the temperature and reached a maximum value at 120 °C or 130 °C. In addition, the proton conductivity increased with the *R* values. As a result, the *R* = 0.95 composite showed the maximum proton conductivity of 9.5 × 10^−4^ S cm^−1^ at 120–130 °C. In contrast, the proton conductivity of the *R* = 0.98 composite abruptly decreased at 130 °C and this value was on the order of 10^−7^ S cm^−1^. This is due to the destruction of the oriented structure, which is assembled by MDP molecules in the composite, through the thermal decomposition of protamine. Next, to evaluate the alkyl chain of MDP, we prepared the P–MP composite, which does not have the long alkyl chain in its molecule, and measured the proton conduction. In this case, the maximum proton conductivity of the P–MP composite was approximately 3 × 10^−5^ S cm^−1^ and this value was more than one order of magnitude lower (see in ✕ Fig. 3). These results suggested that the long alkyl chain of MDP was involved in the formation of the proton conducting pathway. In contrast, the proton conductivity of the P–MP composite was lower than that of the reported value.^11^ This is because the composite used in this experiment is impregnated into the glass filter and the proton conduction includes the resistance of the glass filter.

**Fig. 4:**
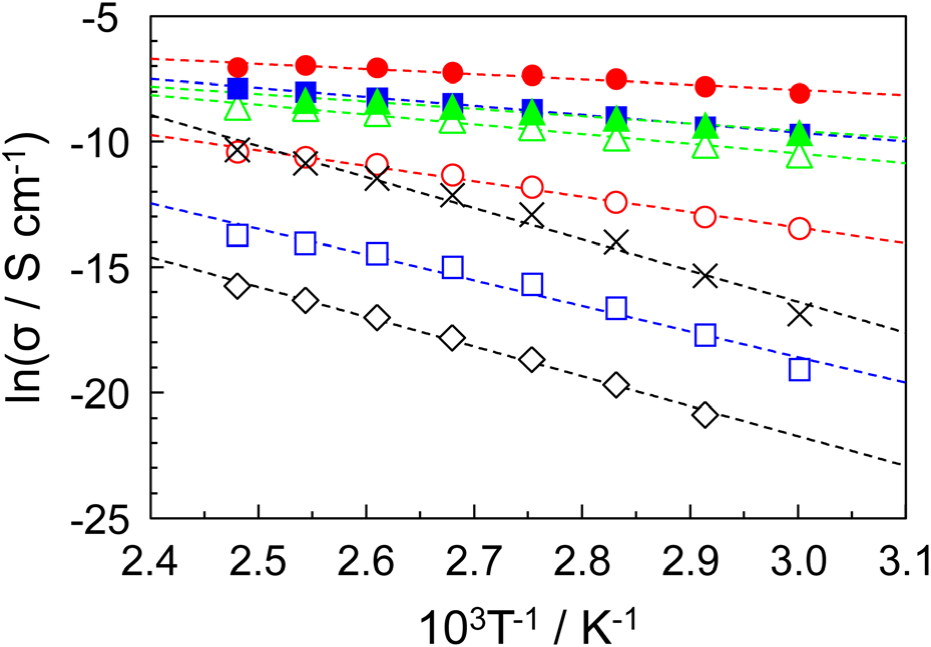
Arrhenius plots of proton conduction of the P–MDP composites. The molar ratios are (◇) *R* = 0.6, (□) *R* = 0.7, (◯) *R* = 0.8, (△) *R* = 0.9, (■) *R* = 0.93, (●) *R* = 0.95, and (▲) *R* = 0.98. (✕) shows the P–MP composite with *R* = 0.95. The straight lines showed the result of a least square fitting. Since the proton conduction for the *R* = 0.98 composite abruptly decreased at 130 °C, this value was excluded from the least square fitting.

The Arrhenius plots of the proton conduction of (◇) *R* = 0.6, (□) *R* = 0.7, (◯) *R* = 0.8, (△) *R* = 0.9, (■) *R* = 0.93, (●) *R* = 0.95, and (▲) *R* = 0.98 composites are shown in Fig. 4. In addition, (✕) in Fig. 4 shows the Arrhenius plots of P–MP composite. Since all the proton conducting samples showed a straight line, no change in the proton conducting mechanism was observed within this temperature range of the individual samples. The activation energy (*E*_a_) based on the proton conduction was calculated from the slope of the straight line.^11,20,30^ These *E*_a_ values are shown in Table 1. The *E*_a_ values decreased with the increased *R* values and showed the minimum value at *R* = 0.95, which indicated the maximum proton conductivity. Generally, the *E*_a_ value of the proton conductor based on the vehicular mechanism is *ca.* 0.02 eV.^21,31,32^ The *E*_a_ values obtained in our experiment were almost one order of magnitude higher than this value. Additionally, the P–MDP composite possesses no mobile ions other than protons in its composite. These results suggested that the proton conducting mechanism of the P–MDP composite was a Grotthuss-type mechanism with the intermolecular proton transfer and the P–MDP composites acted as an anhydrous proton conductor.

**Table 1:** Activation energy (*E*_a_) of proton conduction of the P–MDP composites. The *E*_a_ values were calculated from the slope of the Arrhenius plots

Material	*E*_a_/eV
*R* = 0.6	1.02
*R* = 0.7	0.88
*R* = 0.8	0.53
*R* = 0.9	0.33
*R* = 0.93	0.31
*R* = 0.95	0.18
*R* = 0.98	0.25
P–MP composite	1.07

### 3.4. Construction of proton conducting pathway

**Fig. 5:**
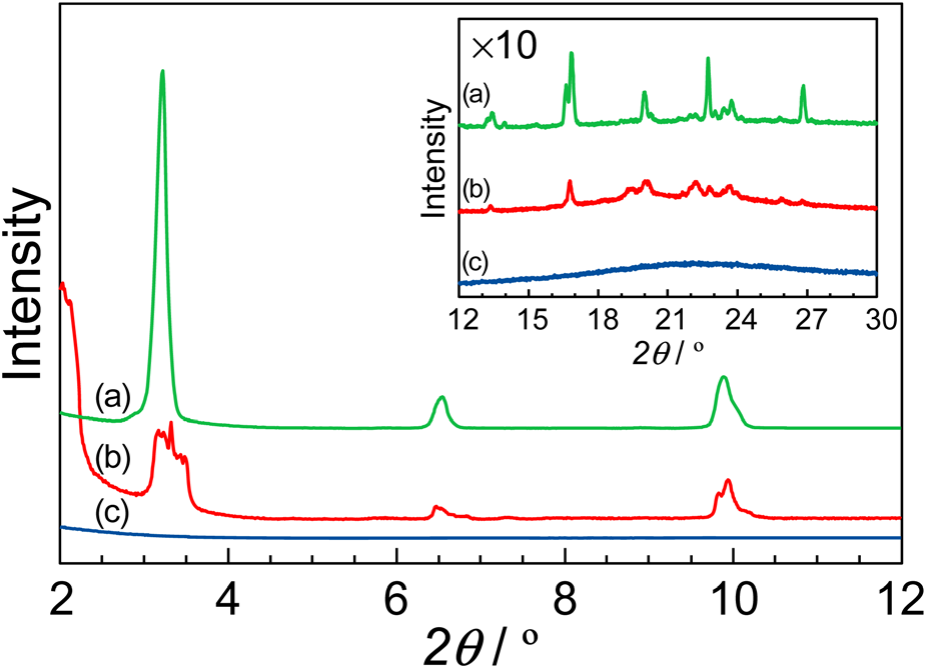
XRD patterns of (a) MDP without the composite, (b) *R* = 0.95 composite, and (c) protamine without the composite in the low angle region. The insert shows the XRD patterns in the high angle region. Similar results were obtained for duplicate experiments.

Fig. 5 shows the XRD patterns of (a) MDP without the composite, (b) the *R* = 0.95 composite, and (c) protamine without the composite in the low angle region. The insert shows the XRD patterns in the high angle region. The protamine without the composite did not show any diffraction pattern. Thus far, it has been reported that the MDP molecule formed a lamellar structure.^33,34^ In our research, the MDP without the composite indicated the diffraction patterns at 3.2°, 6.5°, and 9.9°. These diffraction peaks are related to the lamellar structure which were assigned to (100), (200), and (300). These distances are 27.4 Å, 13.5 Å, and 8.94 Å, respectively, and these distances almost coincided with the reported distances.^33,34^ Additionally, the diffraction pattern at 23.7° appeared as the distance between the alkyl groups and this distance is 3.9 Å. These results suggested that the MDP without the composite formed a lamellar structure with the *d*-spacing of *ca.* 27 Å and the distance between the phosphate groups was *ca.* 4 Å. Similar results were also obtained for the *R* = 0.95 composite. In this case, the *R* = 0.95 composite formed a lamellar structure with the *d*-spacing of *ca.* 26 Å and the distance between the alkyl groups was *ca.* 4.6 Å. For the *R* = 0.95 composite, the values of the *d*-spacing and the distance between the alkyl groups were slightly different from that of MDP without the composite. This is due to the distortion of lamellar structure by the addition of protamine. These results suggested that the MDP with the addition of protamine retained a lamellar structure. Since the phosphate groups in the *R* = 0.95 composite are regularly arranged and the distance between the phosphate groups is *ca* 4.6 Å, these phosphate groups might function as a two-dimensional proton conducting pathway.

### 3.5. Anhydrous proton conductive mechanism of P–MDP composite

Since the MDP can donate two protons and the guanidino group can accept one proton, at *R* = 0.33, all the protons of MDP were deprotonated and all the guanidino groups were protonated. Therefore, P–MDP showed no proton conduction at low *R* values, such as *R* ≤ 0.5. At high *R* values, such as 0.6 ≤ *R*, although all the guanidino groups form the protonated guanidino groups, the phosphate groups exist as a mixture of deprotonated and non-deprotonated phosphate groups. These aggregates of phosphate groups play a role in the proton conductor under anhydrous conditions.

**Fig. 6:**
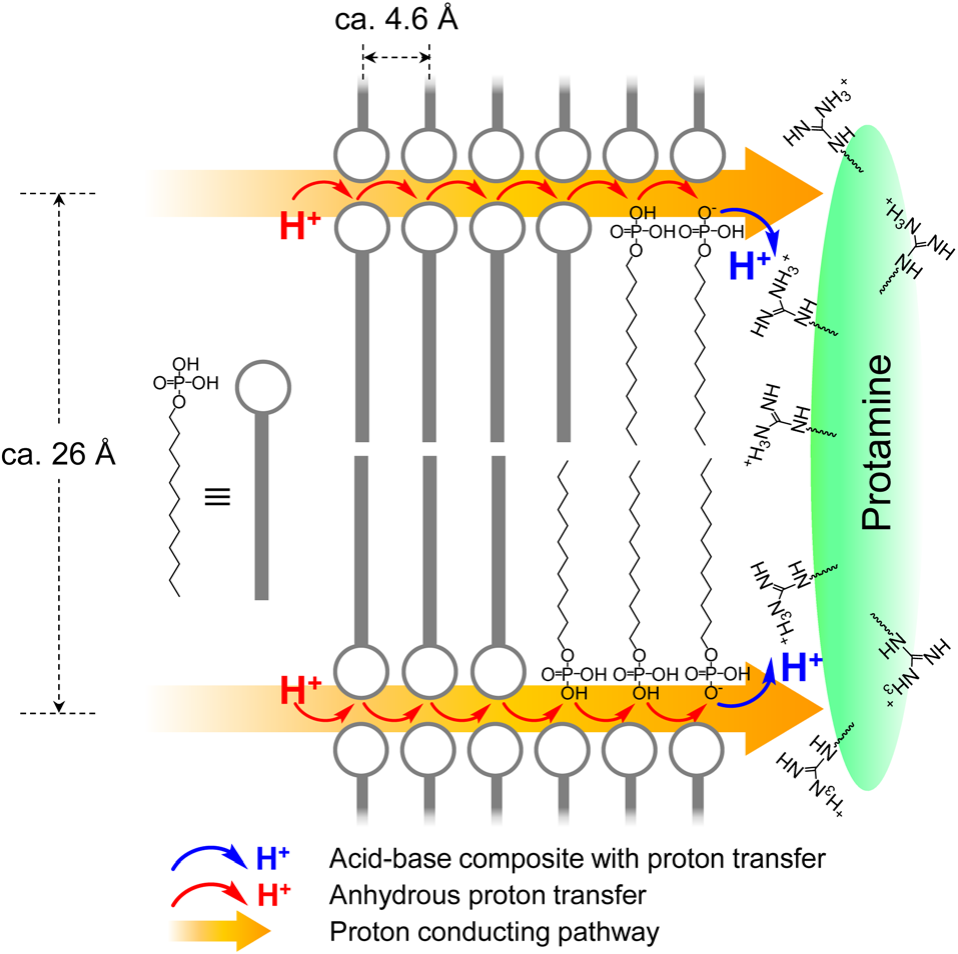
The structural model of the P–MDP composite and a schematic model of the anhydrous proton conduction. The blue arrow and blue proton denote the acid–base composite with the proton transfer. The red arrow and red proton denote the anhydrous proton transfer. The yellow arrows indicate the two-dimensional self-assembled proton conducting pathway.

According to the XRD patterns, the MDP molecule in the P–MDP composite self-assembled and formed a lamellar structure with the *d*-spacing of *ca.* 26 Å. In addition, the distance between the alkyl chains of MDP was *ca.* 4.6 Å. Fig. 6 shows the structural model of the P–MDP composite and the schematic model of the anhydrous proton conduction. The blue and red arrows in Fig. 6 denote the acid–base complex with the proton transfer and the anhydrous proton transfer, respectively. The proton conduction of the P–MDP composite occurred as follows: the phosphate group in MDP formed the deprotonated phosphate group by proton transfer to the guanidino group in the protamine (see blue arrows and blue H^+^ in Fig. 6). At high *R* values, such as 0.6 ≤ *R*, the MDP molecules exist as a mixture of deprotonated phosphate groups, such as P–O^−^, and non-deprotonated phosphate groups, such as P–OH. In addition, since the MDP forms a self-assembled structure in the composites and the distance between the phosphate groups is extremely short, the MDP behaves as a two-dimensional self-assembled proton conducting pathway (see yellow arrows in Fig. 6). Consequently, the proton transfer occurs from the non-deprotonated phosphate group sites, such as P–OH, to the neighboring deprotonated phosphate group sites, such as P–O^−^. Namely, the non-deprotonated phosphate group and the deprotonated phosphate group in the composite can act as a proton donor and a proton acceptor, respectively. Therefore, the P–MDP composite showed the proton conduction of 9.5 × 10^−4^ S cm^−1^ under anhydrous conditions.

## 4. Conclusions

The P–MDP composite was prepared by mixing the protamine (P) and monododecyl phosphate (MDP). These P–MDP composites formed the acid–base composite by electrostatic interactions between the negatively charged phosphate group and positively charged guanidino group. In addition, according to the XRD, the P–MDP composite formed a self-assembled structure along the long alkyl chain. Since the distance between the phosphate groups was extremely short, these MDP molecules acted as a two-dimensional proton conducting pathway. As a result, the P–MDP composite of *R* = 0.95 showed a maximum anhydrous proton conductivity of 9.5 × 10^−4^ S cm^−1^ at 120–130 °C. In contrast, the anhydrous proton conductivity of the P–methanediphosphonic acid (MP) composite, which cannot form a self-assembled structure, was 3 × 10^−5^ S cm^−1^ and this value was more than one order of magnitude lower. By using a protamine, which is discarded as an industrial waste around the world, it is possible to create proton conducting materials at a lower cost than artificial polymers. In addition, the protein, such as protamine, is not only an environmentally benign and biodegradable materials but also a sustainable material. Therefore, the anhydrous proton conductive protein–MDP composite with the lamellar structure has a potential to use for electrodevices, such as biofuel cells and biosensors, in the biomedical, bioengineering, and environmental fields. Furthermore, since the protein composite materials are highly safe for human, they might be applied for the actuators that can be used within the human body.

## Author contributions

Masanori Yamada: conceptualization, data curation, formal analysis, funding acquisition, investigation, methodology, project administration, resources, software, supervision, validation, visualization, writing – original draft, writing – review & editing. Naoaki Yoshihara: formal analysis, software.

## Conflicts of interest

There are no conflicts to declare.

## Supplementary Material

RA-013-D3RA07191J-s001
