# Supplementary material for: Anhydrous proton conductor consisting of protamine–monododecyl phosphate composite with self-assembled structure
Source: RSC Adv. 2023 Nov 30;13(49):34877–83. doi: 10.1039/d3ra07191j (PMC10687518; doi:10.1039/d3ra07191j)
Supplement: RA-013-D3RA07191J-s001 [file RA-013-D3RA07191J-s001.pdf]

Electronic Supplementary Material (ESI) for RSC Advances.
This journal is © The Royal Society of Chemistry 2023

Anhydrous proton conductor consisting of protamine−monododecyl phosphate composite with self-assembled structure

Masanori Yamada* and Naoaki Yoshihara

Department of Chemistry, Faculty of Science, Okayama University of Science, Ridaicho, Kita-ku, Okayama 700-0005, Japan

[*] Corresponding author

Department of Chemistry, Faculty of Science, Okayama University of Science, Ridaicho, Kita-ku, Okayama 700-0005, Japan

Tel: +81 86 256 9550

Fax: +81 86 256 9550

E-mail: myamada@chem.ous.ac.jp

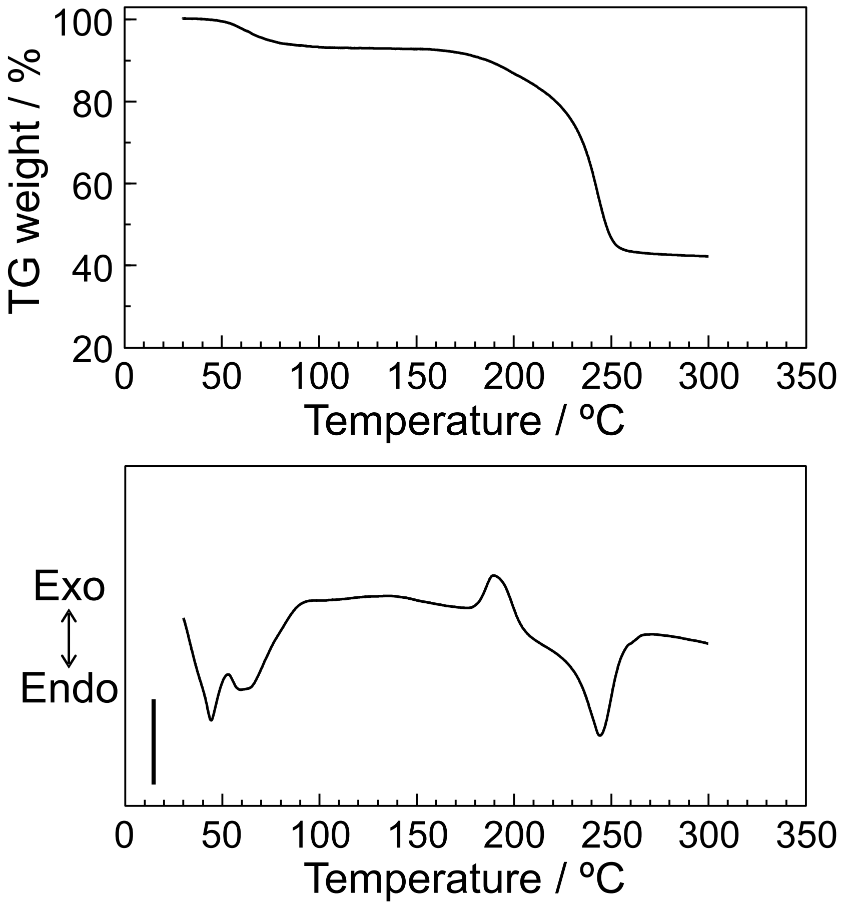

Fig. S1 TG (a) and DTA (b) curves of R=0.95 composite. The TG-DTA measurements were done at the heating rate of 10 °C min^{-1} under flowing dry-nitrogen. The scale bar in Fig. S1(b) shows 20 μV. Similar results were obtained for triplicate experiments.

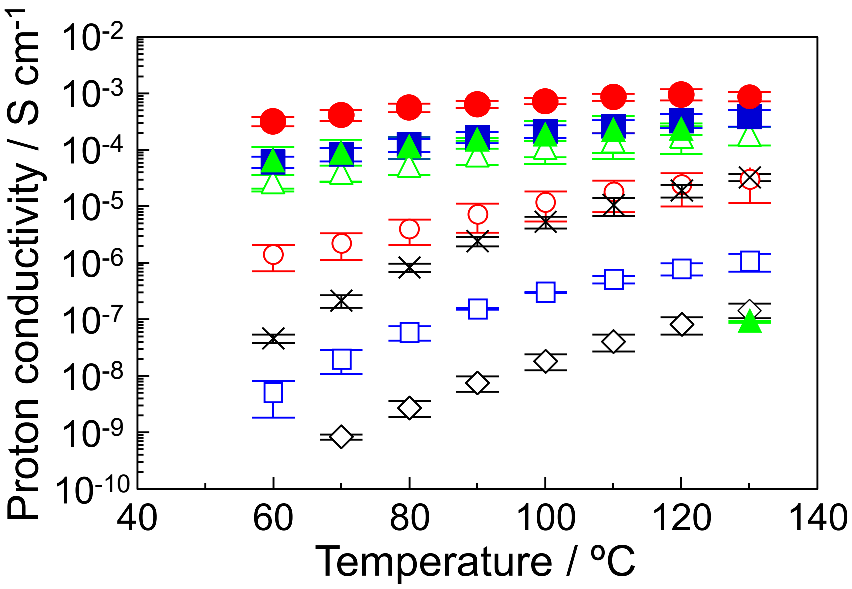

Fig. S2 Proton conductivity of the P−MDP composites. The molar ratios are (❈) *R*=0.6, (⦿) *R*=0.7, (ര) *R*=0.8, (❐) *R*=0.9, (**O**) *R*=0.93, (ഗ) *R*=0.95, and (◘) *R*=0.98. (ℬ) shows the P−MP composite with *R*=0.95. The error bars indicate the standard deviation.
